# The influence of obesity on falls and quality of life

**DOI:** 10.1186/1476-5918-7-4

**Published:** 2008-02-27

**Authors:** Cecilie Fjeldstad, Anette S Fjeldstad, Luke S Acree, Kevin J Nickel, Andrew W Gardner

**Affiliations:** 1 Department of Health and Exercise Science, University of Oklahoma, Norman, OK, USA; 2 Children's Medical Research Institute (CMRI) Metabolic Research Center, University of Oklahoma Health Sciences Center, Oklahoma City, OK, USA

## Abstract

**Objective:**

To determine (1) whether obese older adults had higher prevalence of falls and ambulatory stumbling, impaired balance and lower health-related quality of life (HRQL) than their normal weight counterparts, and (2) whether the falls and balance measures were associated with HRQL in obese adults.

**Methods:**

Subjects who had a body mass index (BMI) greater than 30 kg/m^2 ^were classified into an obese group (n = 128) while those with BMI between 18.5 and 24.9 kg/m^2 ^were included into a normal weight group (n = 88). Functional tests were performed to assess balance, and questionnaires were administered to assess history of falls, ambulatory stumbling, and HRQL.

**Results:**

The obese group reported a higher prevalence of falls (27% vs. 15%), and ambulatory stumbling (32% vs. 14%) than the normal weight group. Furthermore, the obese group had lower HRQL, (p ≤ 0.05) for physical function (63 ± 27 vs. 75 ± 26; mean ± SD), role-physical (59 ± 40 vs. 74 ± 37), vitality (58 ± 23 vs. 66 ± 20), bodily pain (62 ± 25 vs. 74 ± 21) and general health (64 ± 19 vs. 70 ± 18). In the obese group, a history of falls was related (p ≤ 0.05) to lower scores in 4 domains of HRQL, and ambulatory stumbling was related (p ≤ 0.01) to 7 domains.

**Conclusion:**

In middle-aged and older adults, obesity was associated with a higher prevalence of falls and stumbling during ambulation, as well as lower values in multiple domains of HRQL. Furthermore, a history of falls and ambulatory stumbling were related to lower measures of HRQL in obese adults.

## Background

Fatal injuries related to falls are the fifth leading cause of death among older adults in the United States and they are the second leading cause of death due to unintentional injuries [1] and the rates of fall-related deaths seem to be increasing [2]. Obesity is an important factor related to falls in the elderly, as it negatively impacts balance and postural sway, thereby increasing the risk of functional limitations that lead to falling [3]. Fear of falling is also common in older people and is recognized as an important factor for falling in elderly people and is also associated with decreased quality of life [4]. Further, fear of falling is associated with higher levels of physical dysfunction [5], previous falls [6], and suffering from chronic dizziness [7].

Obesity also negatively affects health related quality of life [8-11] possibly due to the development of functional limitations [9,12]. Additionally, obesity can lead to social and economic burdens such as higher risk of depression, lower health related quality of life physical health measures, less likelihood of getting married and having a lower income [13]. When looking at factors associated with falling in healthy elderly people, the use of medication, diseases, body composition and decreased muscle strength were all associated with falling [14]. Increased obesity has shown to positively correlate with impaired postural balance even in younger individuals, less than 40 years of age [15,16]. Further, postural balance was improved in these individuals following weight loss [16] and a weight reduction program lasting as little as three weeks combined with balance training [15]. Studies have examined the influence on obesity on postural stability in the younger generation [15,16], and factors associated with falls in well functional older individuals [14], as well as health related quality of life in middle-aged obese adults [13]. However, little is known about whether poor balance and falls are related to health related quality of life in older obese subjects.

The purposes of this study were to: (a) determine whether obese older adults had higher prevalence of falls, impaired balance, and lower health-related quality of life than their normal weight counterparts, and (b) determine whether falls and balance measures were associated with health-related quality of life in obese adults.

## Methods

### Subjects

#### Recruitment

The subjects participating in this study consisted of 91 males and 125 females, who were recruited through newspaper advertisements, flyers and via mass email message to faculty and staff of the University of Oklahoma.

#### Inclusion and exclusion criteria

Subjects were on average at least 50 years of age who were either of normal weight (body mass index between 18.5 and 24.9 kg/m^2^) or obese (BMI ≥ 30.0 kg/m^2^) were included in this study. Subjects were excluded for the following conditions: (1) being under normal weight defined as a BMI less than 18.5 kg/m^2^, (2) being overweight defined as a BMI between 25.0 kg/m^2 ^and 29.9 kg/m^2^, and (3) neuro-muscular and neuro-physiological diseases determined from a medical history. Each subject signed a written informed consent form and a research privacy form prior to testing. The procedures in this study were approved by the Institutional Review Board at the University of Oklahoma.

### Measurements

Subjects completed a detailed medical history questionnaire to begin the assessment. Height was obtained using a stadiometer, weight was recorded from a physician balance-beam scale, and BMI was calculated as: weight (kg)/height (m^2^). Waist to-hip ratio was obtained using a Gulick measuring tape with horizontal measurements taken at the minimal hip and maximal waist locations.

As previously described [17,18], subjects were asked in writing whether they had fallen over the past year (yes/no). A fall was defined as unintentionally coming to rest on the ground or at some other lower level, not as a result of an overwhelming hazard that would result in a fall by most young, healthy persons [19]. Subjects who reported a fall during the previous year were also asked how many falls they experienced.

Subjects performed a unipedal stance a maximum of 60 seconds and both arms held loosely at their sides [17,18]. This trial required the subject to stand up to 60 seconds on their preferred leg and then on their non-preferred leg. One trial was performed with their eyes opened and one trial with their eyes closed. Additionally, balance was assessed by measuring the time that subjects could stand in tandem, semi-tandem, and full-tandem positions on the floor for a maximum of 10 seconds with their eyes opened and closed [20]. For tandem stance the subject would place their preferred foot in front of the other foot. Any movement of legs off the floor was considered a fall and also anyone that could not complete the 60 seconds or 10 seconds trials. Further, balance was assessed by asking the subjects if they had often stumbled or felt unsteady when they walked over the past year (yes/no). A stumble was defined as a loss of balance that was restored before a fall occurred, and unsteadiness was defined as a routine or regular sense of difficulty with balance while walking [11].

Medical Outcomes Survey Short Form-36 (MOS SF-36) questionnaire was administered to determine health-related quality of life over the previous month [21,22]. This instrument has been widely used and validated in numerous populations [23], and is considered the criterion method in assessing health-related quality of life [24,25]. A total of 36 questions were scored to measure eight domains of health-related QoL. The domains of physical functioning, role limitations due to physical health (role-physical), bodily pain, and general health comprised the physical component of QoL, whereas the domains of vitality, social functioning, role limitations due to emotional health (role-emotional), and mental health comprised the mental component of QoL [25]. Each domain was scored using a scale ranging between 0 and 100, with higher scores indicating a higher QoL than lower scores.

### Statistical analysis

All statistical procedures were performed using SPSS 11.5 for Windows (Chicago, IL). Independent t-tests were done to determine differences between the obese group and normal weight group on the subject characteristics and to assess group differences in balance, falls, and health-related quality of life. Pearson product-moment correlation coefficients were calculated to assess whether balance and falls were related to health-related quality of life within the obese group. All values are expressed as means ± standard deviation (SD), unless stated otherwise. The overall significance was set at p ≤ 0.05 for this investigation.

## Results

Baseline characteristics for the obese and normal weight groups are shown in Table 1. The obese group was significantly older (p = 0.015). The difference in age between the two groups were five years, 60 yrs vs 55 yrs between obese and non-obese controls respectively. Further, the obese group had greater weight (p < 0.001), BMI (p < 0.001) and waist-to-hip ratio values (p < 0.001). The groups were similar in height (p > 0.05), but the obese group having greater prevalence (p > 0.05) of hypertension, arthritis, diabetes and hyperlipidemia (Table 2).

**Table 1:** Subject characteristics in obese and normal-weight groups. Values are in means (SD).

Variables	Obese (N = 128)	Non-Obese (N = 88)	P Value	Effect Size	Estimated Power
Age (yrs)	60 (12)	55 (17)	0.015	.35	.74
Height (cm)	168 (10)	166 (8)	0.117	.22	.38
Weight (kg)	100.5 (21)	63.5 (10.6)	< 0.001	2.28	~1.0
Body Mass Index	35.0 (6.7)	22.8 (3)	< 0.001	2.44	~1.0
Waist-to-Hip Ratio	0.88 (0.09)	0.81 (0.09)	< 0.001	.78	~1.0

**Table 2:** Subject characteristics in obese and normal weight groups. Values are in percentages.

Variables	Obese (N = 128)	Non-obese (N = 88)
Hypertension (%)	51	23
Arthritis (%)	50	31
Diabetes (%)	13	4.5
Hyperlipidemia (%)	48	31
Fall history (%)	27	15
Stumbling (%)	32	14

The obese group had a higher prevalence of falls (27% vs. 15%) and ambulatory stumbling (32% vs. 14%) than the non-obese group respectively (Table 2). No group differences (p > 0.05) were found for any of the standing balance tests (Table 3).

**Table 3:** Balance measures in obese and normal weight groups. Values are in means (SD).

Variables	Obese (N = 128)	Non-Obese (N = 88)	P Value	Effect Size	Estimated Power
Side by Side Stance: Eyes Open (sec)	10.00 (0)	10.00 (0)	0.900	------	------
Side by Side Stance: Eyes Closed (sec)	9.90 (0.78)	9.91 (0.78)	0.232	.01	.10
Semi Tandem Stance Eyes Open (sec)	9.95 (0.45)	10.00 (0)	0.497	.22	.38
Semi Tandem Stance Eyes Closed (sec)	9.74 (1.3)	9.62 (1.3)	0.605	.09	.15
Tandem Stance: Eyes Open (sec)	8.90 (2.4)	9.07 (2.3)	0.798	.07	.13
Tandem Stance: Eyes Closed (sec)	6.79 (3.4)	6.91 (3.5)	0.538	.04	.08
Unipedal Stance: Eyes Open (sec)	27.06 (24.7)	29.21 (25.0)	0.706	.09	.15
Unipedal Stance: Eyes Closed (sec)	6.30 (10.8)	6.91 (12.4)	0.538	.05	.09

EO = eyes open and EC = eyes closed.

The obese group had lower quality of life measurements in the following five domains; physical function (p = 0.002), role physical (p = 0.004), vitality (p = 0.017), bodily pain (p < 0.001), and general health (p = 0.016). The groups were similar (p > 0.05) in the role-emotional, mental health and social function domains of health-related quality of life measurements (Table 4).

**Table 4:** Health-related quality of life measures in obese and normal weight groups. Values are means (SD).

Variables	Obese (N = 128)	Non-obese (N = 88)	P Value	Effect Size	Estimated Power
Physical Function	63 (27)	75 (26)	0.002	.45	.90
Role-Physical	59 (40)	74 (37)	0.004	.39	.84
Role-Emotional	81 (32)	79 (35)	0.777	.06	.10
Vitality	58 (23)	66 (20)	0.017	.37	.79
Mental Health	78 (16)	79 (14)	0.730	.07	.13
Social Function	81 (22)	85 (20)	0.239	.19	.35
Bodily Pain	62 (25)	74 (21)	<.001	.52	.96
General Health	64 (19)	70 (18)	0.016	.32	.64

In the obese group, a history of falls was related to shorter times to maintain balance in the side-by-side stance with eyes closed (p < 0.01), and in the semi-tandem stance with eyes closed (p < 0.05). Further, a history of falls was related to lower health-related quality of life domains (Table 5) of social function (p < 0.05), bodily pain (p < 0.05), physical function (p < 0.05) and role limitations due to physical health (p < 0.01), and a history of ambulatory stumbling was related to lower health-related quality of life in all domains (p < 0.01). No relationship was shown between history of falls and role limitations due to emotional health (p > 0.05). While the correlations are statistical significant, they were overall generally small with the highest at 0.45, which could be due to the large sample size.

**Table 5:** Correlation coefficients between fall and balance measures and health-related quality of life in obese subjects.

Variables	Fall History	Stumbling
Physical Function	-0.204*	-0.401**
Role-Physical	-0.249**	-0.258**
Role-Emotional	-0.006	-0.097
Vitality	-0.081	-0.362**
Mental Health	-0.155	-0.299**
Social Function	-0.202*	-0.238**
Bodily Pain	-0.210*	-0.450**
General Health	0.016	-0.283**

** Correlation coefficient significant at the 0.01 level (2-tailed), * 0.05 level (2-tailed)

## Discussion

This investigation examined standing balance, prevalence of falls and ambulatory stumbling, and health-related quality of life between obese and normal weight older groups, and whether the falls and balance were related to quality of life in obese subjects. The primary findings were that the obese group had a higher prevalence of falling and ambulatory stumbling, as well as lower quality of life in multiple health domains than their normal weight counterparts. The secondary finding was that a history of falls was related to lower measures of quality of life in obese men and women.

The increased risk of falling and fear of falling have long been recognized as common problems in older individuals [4,26], but few studies have investigated the inter-relationship among falling, obesity and quality of life. Our findings that obese adults are at greater risk of falling a aagree with previous studies [27,28]. Over one-quarter of the obese subjects in this study reported a history of falling, whereas only 15% of the normal weight group had a history of falling. A study done by Corbeil et al. [27] suggested that obese people with abnormal distribution of body fat, particularly in the abdominal area, might be at an increased risk of falling compared to individuals who are not obese. The greater risk of falling in the obese group corresponds with their greater prevalence of ambulatory stumbling, as nearly one-third of the obese group had a history of stumbling compared to only 14% in the normal weight group. This finding supports a previous study that showed obese individuals had inadequate postural stability compared to their non-obese counterparts [15]. Interestingly, the measures of standing balance were not different between the groups in our study, suggesting that falling in obese subjects may be more related to a dynamic measure of balance, such as ambulatory stumbling, than to static measures of balance while standing.

Obese individuals are typically sedentary as there is an inverse relationship between BMI and activity levels [28]. An increase in BMI is not only negatively associated with physical activity levels, but it is also associated with an increase in functional impairment [3], which could possible lead to impaired balance and an increased risk of falls. Consequently, obese individuals may fear f alling, which may lead to further reduction in physical activity [28], greater functional impairment [4], and greater risk of falling. Activity programs including resistance training, stretching and an increase in balance confidence have shown to decrease the fear of falling and thus have a positive impact on the elderly [29-31].

The current investigation found that obese adults have impaired quality of life in five of the eight health domains. These findings are supported by a previous investigation, which found obesity was inversely associated with health-related quality of life and functional measures in middle-aged individuals [13]. Furthermore, the present study reports that obesity has a greater impact on the physical component of quality of life than on the mental component. Specifically, all four domains comprising the physical component of quality of life were lower in the obese group than in the normal weight group, whereas only the vitality domain was impaired in the mental component of quality of life. This is consistent with previous work in which women with high BMI values had significant lower scores on the physical components of quality of life compared to the mental components [13]. Additionally, obese individuals have poorer self-reported health and more days of poor health compared to their non-obese counterparts [9].

Obese older adults may experience decreased quality of life because excess body fat can interfere with daily activities of physical functioning, such as walking, bending, stooping and kneeling [32]. A decreased ability to perform these physical tasks can possibly lead to dependency on other individuals for aid with daily household chores, [33]. Consequently, obese individuals may feel a sense of inadequacy or failure that may lower their quality of life in multiple domains in both physical and mental health.

The primary limitation to this study is the cross-sectional design, which establishes the association that obesity has with balance, falls, and quality of life. Although it is possible that obesity leads to impairment in these factors, it is equally plausible that obesity is a consequence of them. Prospective, longitudinal or interventional studies that measure weight gain or weight loss are necessary to better understand the role that obesity has on balance, falls, and quality of life. Another limitation to this study is the self-report nature of many of the outcome measures, such as the assessment of quality of life, and history of ambulatory stumbling and falls. Further, another limitation to this study is the use of medication, which was not controlled for. Some medications frequently used in the older population could possible influence postural balance. Vestibular function, problems with eyes or ears, and the use of alcohol are other factors that could influence balance that were also not controlled for. There was a five-year mean age difference between the non-obese and the obese group in this study that may impact the results and is a major limitation. This is a very large variability with a range in age of 19–85 years. So, there is a chance that the differences seen in hypertension, arthritis, diabetes and hyperlipidemia could be due to the range in age as it is more commonly seen in older people rather than by obesity alone. A final limitation is that there may be underlying conditions that were not measured, such as depression, that partially contribute to the association between obesity, balance, and falls.

In middle-aged and older men and women, obesity was associated with a higher prevalence of falls and stumbling during ambulation, as well as lower values in multiple domains of health-related quality of life. Furthermore, a history of falls and ambulatory stumbling were related to lower measures of quality of life in obese men and women. Future studies appear warranted to assess whether weight loss interventions improve quality of life and reduce the risk of falling in older obese adults.

## Competing interests

The author(s) declare that they have no competing interests.

## Authors' contributions

CF acquired the data, recruited subjects, worked on the statistical analyses of the manuscript and the interpretation of the data, as well as drafting and revising the manuscript. ASF, KJN, and LSA all recruited subjects and revised the manuscript. AWG designed the study, assisted with both statistical analyses and the interpretation of it, and also drafted and revised the manuscript. The final draft of the manuscript was read and approved by all authors.
